# Bystanders witnessing social exclusion exhibit reduced physiological responses of empathy: an EEG study

**DOI:** 10.3389/fpsyg.2025.1593873

**Published:** 2025-12-10

**Authors:** Satsuki Torige, Shinobu Yasumuro, Sunao Iwaki

**Affiliations:** 1 Graduate School of Comprehensive Human Sciences, University of Tsukuba, Tsukuba, Ibaraki, Japan; 2 Human Informatics and Interaction Research Institute (HIIRI), National Institute of Advanced Industrial Science and Technology (AIST), Tsukuba, Japan

**Keywords:** bystander, empathy, EEG, bullying, theta band power

## Abstract

Bystanders play a crucial role in bullying interventions, with empathy serving as a key facilitator of bystanders’ helping behaviors toward victims. However, the physiological evidence linking bystanders’ empathic responses to prosocial behaviors is limited. In addition, differences in empathic responses between active and passive bystanders have not been investigated, although bystanders have been suggested to disengage from bullying depending on the situation. This study aimed to reveal how the involvement of bystanders modulates empathic responses while witnessing social exclusion using electroencephalography (EEG), specifically focusing on the changes in the amplitudes of frontal theta and alpha band spontaneous activities as indicators. Participants engaged in an Inclusion and Exclusion condition in two sessions of the Cyberball task: witness only and witness in participation. The results showed a significant main effect of the condition on theta band power (TBP), with a decrease in TBP while witnessing exclusion. Generally, the frontal theta power is amplified as an empathic response. However, the current results indicate that frontal theta power decreased by observing social exclusion, suggesting that, regardless of bystander situations, bystanders witnessing bullying may protect themselves by decreasing empathy or moral conflict.

## 1 Introduction

Bullying has increased globally in recent years. A large cross-national study of 15-year-olds across 71 countries reported that 30.4% experienced frequent victimization (Hosozawa et al., 2021). Bullying has been defined in multiple ways; for example, Volk et al. (2014) describe it as “aggressive, goal-directed behavior that harms another individual within the context of a power imbalance.” Cyberbullying—occurring online or in virtual spaces—has been broadly defined as “willful and repeated harm inflicted through computers, cell phones, and other electronic devices” (Hinduja and Patchin, 2015). Both bullying and cyberbullying impose significant psychological and health costs on victims (Wolke et al., 2013; McLoughlin et al., 2020), and high-profile cases of online slander have tragically been linked to suicide.

It is essential, however, to distinguish bullying, social exclusion, and social pain, which are related but non-identical constructs. Social exclusion refers to being prevented from participating in social interactions and can occur without bullying—for example, when group dynamics lead to being left out without aggressive intent. Conversely, bullying can occur without exclusion (e.g., direct verbal or physical aggression while the victim remains socially included). Social pain denotes the aversive affective response to rejection or ostracism (Eisenberger, 2012) and does not, by itself, imply bullying. Clarifying these distinctions is crucial for theorizing empathic responses to different forms of social adversity; the present study focuses specifically on empathy for observed social exclusion.

Neuroimaging work has delineated brain systems implicated in victims’ experiences of exclusion, including the right ventral and left ventrolateral prefrontal cortices, bilateral hippocampus, left middle temporal gyrus, dorsal anterior cingulate cortex (dACC), and ventral striatum (Eisenberger, 2012; Masten et al., 2012; Bolling et al., 2011). Younger adolescents show functional alterations in prefrontal, midline, and limbic regions during exclusion (Vijayakumar et al., 2017). While the anterior cingulate cortex (ACC)—particularly dACC and ventral ACC (vACC)—is reliably linked to social pain (Rotge et al., 2015), some studies suggest ACC responses may reflect expectancy violation (Somerville et al., 2006; Bolling et al., 2011).

Complementing fMRI, EEG offers millisecond-level temporal resolution for tracking information processing (Liu et al., 2020). A systematic review (Mills et al., 2024) synthesizing power and ERP findings reported that social exclusion is associated with increased N200 (≈200 ms; deviance detection) and P300 (≈300–800 ms; memory/attention) (Sur and Sinha, 2009), as well as more negative frontal late slow waves (evaluation; Crowley et al., 2010) and alterations in frontal theta activity linked to distress/anxiety. Longitudinal evidence further suggests that resting-state spectral profiles may index roles in cyberbullying across adolescence, underscoring the developmental value of EEG markers (e.g., Mills et al., 2025).

Despite these advances in understanding victims, neuroscientific work on bystanders—who are present in most incidents and can shape outcomes—is comparatively limited (Hawkins et al., 2001; Mazzone, 2020). Bystanders may alleviate bullying by helping victims or preventing escalation, yet passivity can inadvertently reinforce perpetrators (Saarento and Salmivalli, 2015; Salmivalli et al., 1996). Passive bystanders increase victims’ negative affect and reduce subsequent helping (Staub et al., 2003), and exposure to passivity can dampen intentions to support victims (Howard et al., 2014), potentially signaling tacit approval (Kärnä et al., 2011). These behavioral dynamics motivate a closer look at the empathic mechanisms that drive (or inhibit) prosocial intervention.

Empathy is a key driver of prosocial responding (Eisenberg and Miller, 1987; van Noorden et al., 2015; Riess, 2017). We distinguish affective (emotional) empathy—sharing or resonating with others’ feelings—from cognitive empathy—inferring and understanding others’ mental states. We use mentalization to denote the ability to understand others’ behavior in terms of their mental states, including beliefs, intentions, and emotions (Frith and Frith, 1999). Low empathy is associated with aggression and indifference (Decety, 2010) and relates positively to bullying perpetration (Foshee et al., 2016; Xiong et al., 2023). Meta-analytic and systematic reviews indicate a negative association between bullying and empathy—particularly affective empathy—and link higher empathy to defending behavior (van Noorden et al., 2015; Deng et al., 2021; Hikmat et al., 2024). School-based programs that target bystanders’ empathy and self-efficacy (e.g., KiVa) reduce victimization and perpetration (Bezerra et al., 2023). Recent evidence suggests that affective empathy, together with personal and peer attitudes, robustly predicts prosocial bystander intervention, with cognitive empathy showing smaller, less consistent effects (LaChance et al., 2025).

At the neural level, fMRI studies implicate ACC, middle cingulate cortex, and anterior insula (AI) in affective empathy, and dorsomedial/ventromedial PFC, temporoparietal junction, superior temporal sulcus, and temporal poles in cognitive empathy (Stevens and Taber, 2021). EEG studies converge on frontal midline theta (originating in ACC/mPFC; Asada et al., 1999; Ishii et al., 1999) as indexing the interplay of cognitive control and affective resonance during empathy (Mu et al., 2008; Han, 2018; Lavin et al., 2023; Romeo and Spironelli, 2024). For instance, a study on young adults also found that enhanced empathy and norm compliance, elicited by perceiving another person in need, were associated with increased frontal-central theta activity (Lavin et al., 2023). This suggests that theta synchronization reflects the recruitment of cognitive control and affective resonance processes during empathic engagement. In particular, frontal midline theta, originating from the anterior cingulate cortex (ACC) and medial prefrontal cortex (mPFC) (Asada et al., 1999; Ishii et al., 1999), is consistent with fMRI findings showing that these regions mediate the integration of affective and cognitive components of empathy (Morrison et al., 2004; Jackson et al., 2005; Masten et al., 2011). Empathic processing is typically accompanied by alpha and mu suppression, reflecting increased sensorimotor resonance with observed others (Perry et al., 2010; Whitmarsh et al., 2011; Motoyama et al., 2017; Yang et al., 2009; Woodruff et al., 2011; Fan et al., 2014). These decreases in oscillatory power are interpreted as neural markers of enhanced mirroring and emotional engagement during empathy.

Extending to bystanders, fMRI work shows that witnessing exclusion engages mentalizing regions (dmPFC, mPFC, precuneus) and, in highly empathic individuals, co-activates social-pain–related regions (AI, dACC), with activity relating to prosocial behavior (Masten et al., 2011; McIver et al., 2022). ERP studies of vicarious ostracism report frontal feedback-related negativities and posterior late positive potentials (LPP) associated with compassion and victim-helping (Rodriguez et al., 2023). Nevertheless, physiological indices of bystanders’ empathy remain under-characterized.

Addressing this gap, we investigate the neural dynamics of empathy while witnessing social exclusion and their relationship to bystander behavior. We employ a modified Cyberball paradigm (Williams et al., 2000; Eisenberger et al., 2003; Ueda et al., 2021) with a within-subjects structure comprising (i) a witness-only (WO) session that isolates observational processing (no means to intervene) and (ii) a witness-in-participation (WIP) session that permits opportunity to act (to help the excluded player, P3). This structural separation avoids confounding voluntary non-intervention with mere absence of opportunity and provides an initial test of whether empathic responses depend on opportunity to act. Both sessions included inclusion (IC) and exclusion (EC) blocks, enabling us to compare neural and behavioral responses under both social contexts. Given the temporal limitations of BOLD responses (~3 s width; peak at 5–6 s; Glover, 2011), we use EEG to capture fast neural dynamics, focusing on frontal theta power and alpha/mu suppression as candidate indices of empathic engagement.

This study tested the following hypotheses.

1. Based on previous findings, witnessing social exclusion elicits distinctive neural signatures of empathic processing: observers show increased frontal theta activity and alpha suppression when witnessing social exclusion, regardless of their ability to intervene.
2. When intervention is not possible, the prolonged observation of social exclusion leads to enhanced neural responses. In the non-intervention condition, stronger frontal theta amplification and greater alpha suppression will be observed compared to those who cannot intervene, reflecting increased empathic processing of the exclusionary situation.
3. Neural markers of empathic responses significantly correlate with prosocial behavior. Specifically, greater EEG responses to observed social exclusion are associated with increased helping behavior toward victims.

## 2 Methods

### 2.1 Participants

Eighteen healthy paid volunteers (six females and 12 males) participated in the study. The mean age was 23.1 ± 3.6 years. All participants provided written informed consent before the experiment. All the experimental methods and procedures were approved by the Internal Ethics Committee of the National Institute of Advanced Industrial Science and Technology (approval #2022-799A).

### 2.2 Experiment design

This experiment used a 2 × 2 within-subjects design. Participants performed the Cyberball task in two sessions: Include condition (IC) and Exclude condition (EC). Four players were shown on the screen: Player 2 (P2), operated by the participant, and three other computer-programmed players (P1, P3, P4) participated in the task. In the IC, the computer-operated players randomly exchanged passes with all players, whereas in the EC, P1/P4 excluded P3 from the game.

In this study, to emphasize the empathic responses induced by observing exclusion, participants were taught about the relationships among other players in the task before the IC/EC started. However, prior instruction about these relationships could influence participants’ behavior. To minimize this potential influence, direct expressions such as “bullying” or “exclusion” were avoided, aiming to reduce any unintended behavioral impact. Afterward, they were asked to watch other players catch up without participating themselves (witness only session; WO). They then participated in the cyberball task (witness in participation session; WIP). In the WO condition, participants had no opportunity to intervene and could only observe the exclusion. In the WIP condition, participants could help the excluded player by choosing to throw the ball to P3, allowing us to distinguish choice-based passivity (throwing to excluders) and active defending (throwing to the excluded player).

Importantly, the experimental sequence was fixed: participants always completed IC before EC. This procedure was adopted based on findings demonstrating short-term adaptive changes on empathy-related neural activity by preceding emotional stimuli. Specifically, Mu and colleagues showed that theta and alpha oscillations associated with empathic responses significantly decreased after exposure to preceding emotional stimuli, indicating rapid neural adaptation within experimental sessions (Mu et al., 2008) Given that empathy can adapt during brief tasks and that witnessing bullying may reduce subsequent empathic responses (Pabian et al., 2016)conducting EC first could induce carryover effects that would compromise the measurements of baseline empathic responses in the IC trials. By administering IC first, we sought to obtain a baseline empathic responses prior to exposure to exclusion. Nevertheless, we acknowledge that this fixed order may introduce systematic order effects due to task familiarity or changes in participants’ state over time, which represent a limitation of the current experimental design.

The participants used the computer keyboard to select which player to throw the ball at each time they received it in the WIP session. The total number of trials in the WIP session was 150, and both conditions were conducted in two sessions. The WO session consisted of 60 trials.

### 2.3 Stimuli and procedure

**Figure 1:**
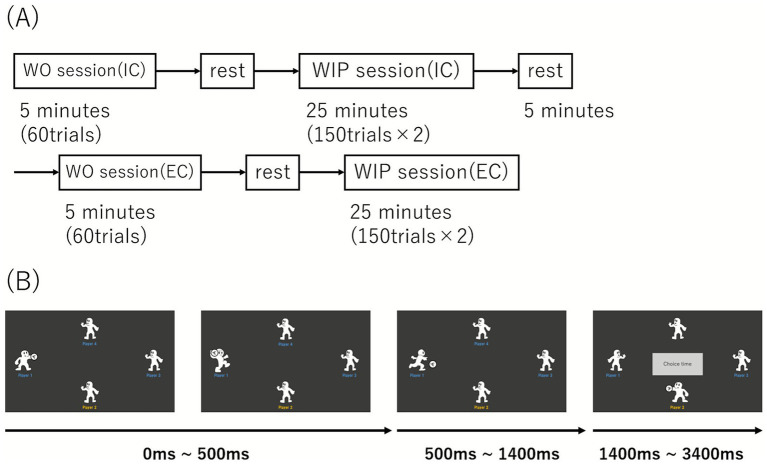
**(A)** This figure illustrates the experimental procedure. After completing the witness-only (WO) and witness-in-participation (WIP) sessions in the Inclusion condition (IC), participants performed the same sequence of tasks in the Exclusion condition (EC). **(B)** Temporal sequence and structure of the Cyberball task. The trial sequence started with target selection, followed by throwing motion and ball release after 500 ms. Time to receiver was fixed at 900 ms. The total time from target selection to ball reception was 1,400 ms. When participant participants (P2) received the ball, a 2,000 ms “Choice time” display appeared in the center of the screen for target selection. In the Exclusion condition, P1 (left) and P4 (top) acted as the excluders, while P3 (right) was the excluded player (victim).

In this experiment, to evaluate empathic responses using EEG and the relationship between empathy as a personal trait (measured by self-report) and behavior, we used the 24-item Multidimensional Empathy Scale (MES; Suzuki and Kino, 2008). We adopted the MES because it provides a culturally validated instrument with five theoretically coherent subscales that together cover both affective and cognitive facets of empathy This questionnaire consists of five factors: (a) Other-Oriented Emotional Reactivity, (b) Self-Oriented Emotional Reactivity, (c) Emotional Susceptibility, (d) Perspective Taking, and (e) Fantasy; participants responded on a 5-point Likert scale (1 = Strongly Agree, 5 = Strongly Disagree). The 24-item Multidimensional Empathy Scale (MES; Suzuki and Kino, 2008) was administered once before the EEG session to assess trait empathy. We originally intended to examine trait empathy as a potential covariate to account for individual differences in prosocial responding. Given the high intercorrelations among MES subscales and the limited sample size (*N* = 18), we used the total score (averaged across all items) as a global index. Preliminary Pearson’s correlation analysis revealed no significant association between MES total scores and the behavioral index (ΔP3 throws; TBP (*r* = −0.26, *p* = 0.29) and ABP (*r* = −0.03, *p* = 0.90)). Based on this finding and to avoid overfitting with our small sample, we did not include trait empathy as a covariate in the main ANOVA models. Individual subscale scores were not analyzed separately. The Cyberball task used in the experiment was created using Inquisit Lab software (Millisecond Software, LLC, Seattle, WA, USA). For visual stimuli, a 25-inch screen with a resolution of 1,920 × 1,080 pixels was used. Participants viewed it from a distance of 60 cm. Thus, the visual angles were approximately 28.1° (vertical) and 47.7° (horizontal). The Cyberball task began with a throwing motion, and the ball was released after 500 ms. The time for the ball to reach the receiver was fixed at 900 ms. Thus, the total time from target selection to ball reception was 1,400 ms. The player who received the ball entered the target selection phase. Computer-programmed players were set to initiate their next throwing motion at random intervals of either 2,000 or 3,000 ms. When the participant received the ball, a “Choice Time” display appeared in the center of the screen for 2000 ms to allow target selection (Figure 1). The participants selected the throwing target using a numeric keypad (ELECOM, TK-TCM011). In the Exclusion condition, P1 (left) and P4 (top) acted as excluders, whereas P3 (right) was treated as the excluded player (victim).

Before the experiment, participants were given written and verbal instructions regarding the task requirements. After completing the 24-item Multidimensional Empathy Scale (Suzuki and Kino, 2008), participants participated in a practice session with 20 trials to ensure that they could perform the task as instructed. In the formal experiment, participants received the following information before the IC started, “You are about to join a catch ball game. The interpersonal relationships are good.” After the WO session, two sessions (WIP) of 150 trials were conducted in the IC. Subsequently, the EC was conducted using the same procedure. Prior to the start of the EC, participants received the following information: “You are now joining the catch ball game. Relationships among the other members are not good. Specifically, Player1/4 will not pass to Player3.” Participants were asked to consider social relationships during the experimental trials and to choose the player to throw to within 2 s of receiving the pass. Participants could not throw the pass during “Choice time.” Participants were given a minimum of 2 min of rest at the end of each session (5 min between IC and EC), and they could extend the rest time based on their tiredness level. They were also asked to minimize blinking and eye movement throughout the experiment to reduce EEG artifacts.

### 2.4 EEG recordings

EEG activity was recorded using an amplifier (BrainAmp Standard; Brain Products GmbH, Germany) and an active EEG electrode system (actiCAP slim; Brain Products GmbH, Germany). The EEG system used 19 electrode sites (Fp1, Fp2, F7, F3, Fz, F4, F8, T7, C3, Cz, C4, T8, P7, P3, Pz, P4, P8, O1, and O2), and reference and ground electrodes. Horizontal and vertical electrooculograms (EOG) were recorded simultaneously using electrodes placed on the outer left and right canthi and above and below the right eye. The impedance of all the electrodes was maintained below 25 kΩ. The EEG signals were digitized at a sampling rate of 1,000 Hz. EEGLAB (Delorme and Makeig, 2004) was used for data preprocessing and subsequent analysis. Preprocessing was performed to clean the raw EEG data and increase the signal-to-noise ratio of the movable EEG datasets. The EEG signals were re-referenced to the averaged EEG signals from the linked earlobes (A1 and A2) and bandpass filtered at 1–45 Hz. The artifacts derived from the EOG and body movements were removed from the data using independent component analysis. To compute the event-related spectral perturbation (ERSP) elicited by the target stimuli, epochs ranging in time from −1,500 ms to 2,500 ms were extracted for all trials between P1 and P4, which were the focus of the analysis.

### 2.5 Data analysis

The preprocessed data were analyzed using MATLAB (MathWorks, Natick, MA, USA). The primary behavioral measure was the rate of throws to P3, who was excluded from the game. Owing to the randomized throw sequence, the participants’ total number of throws varied between conditions. The throw rate was calculated for each condition by dividing the number of throws to P3 by the total number. A paired t-test was used to compare differences in throw rates between IC and EC conditions. Pearson’s correlation analysis was performed with self-reported empathy and the amount of change in throw rate to examine the relationship between empathy and behavior. The change in throw rate to P3 was calculated by subtracting the throw rate in the IC from that in the EC.

EEG time-frequency representation (TFR) was calculated using wavelet decomposition with a Morlet wavelet basis. Time-frequency power values were normalized to the baseline period (−1,500 ms to 0 ms) to represent the power ratio between the target and baseline period activities.

In this study, six frontal electrodes (F3, Fz, F4, C3, Cz, and C4) were selected (and averaged) to examine the frontal ERSP. Two frequency bands were analyzed: (a) TBP (4–8 Hz) and (b) alpha band power (ABP) (8–12 Hz). In each frequency band, the power was averaged over the 1,000 ms window (from 500 ms to 1,500 ms post-throw), corresponding to the time during which the ball traveled between the thrower and catcher. A 2 × 2 repeated-measures ANOVA was conducted with task (WO vs. WIP) and condition (IC vs. EC) as within-subject factors. Significant ANOVA effects were followed up with post-hoc t-tests. Pearson’s correlation analysis was performed on behavioral data and TBP/ABP during the WIP to examine the relationship between behavior and EEG responses.

## 3 Results

### 3.1 Behavioral analysis

**Figure 2:**
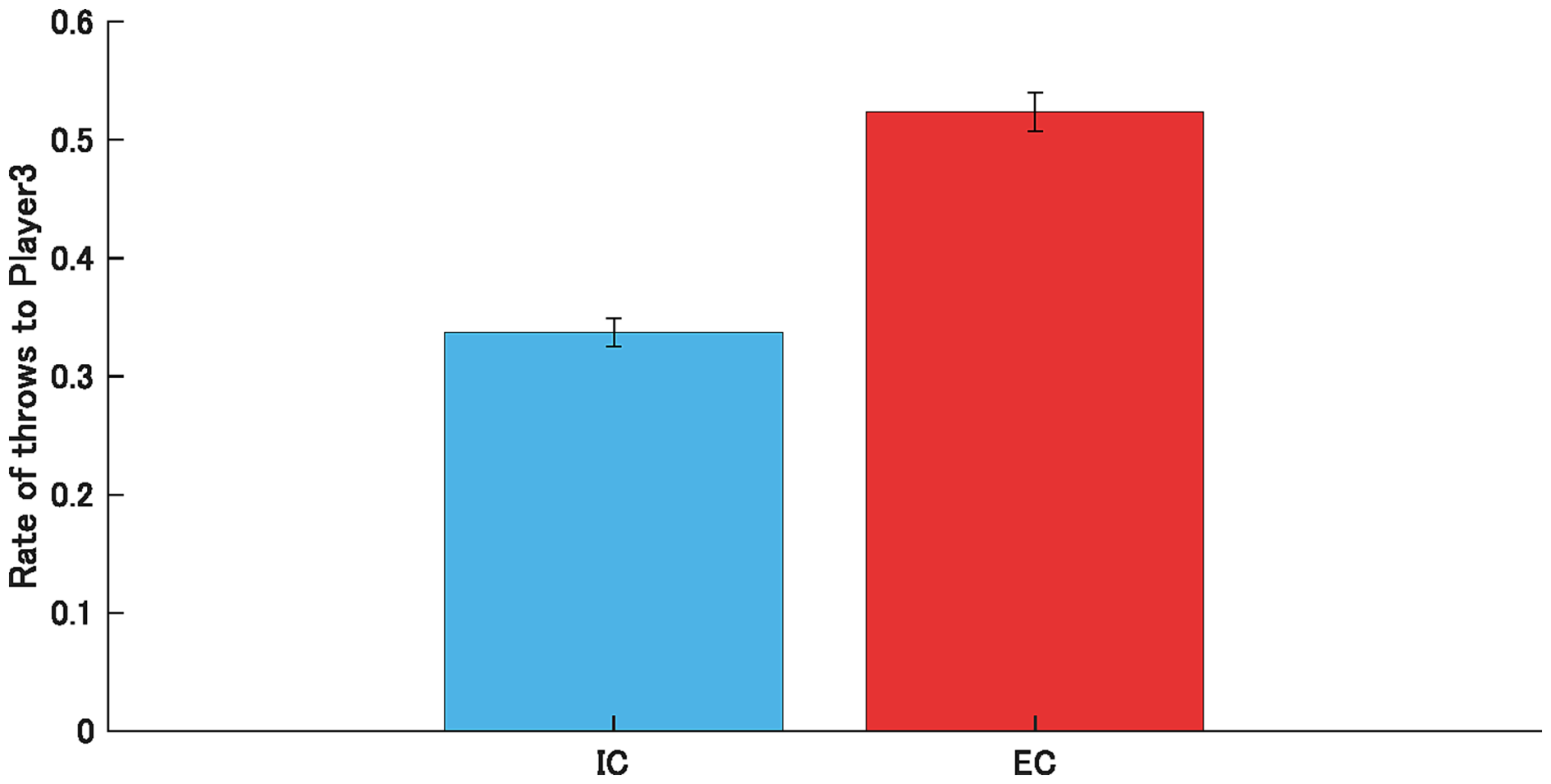
Average throwing rate to Player 3 in the inclusion condition (IC) and the exclusion condition (EC), with error bars representing the standard error (SE) across participants. Throw rate to P3 was significantly increased in EC (*p* < 0.001).

Figure 2 shows the average throw rate to P3 under the IC and EC. The results showed a significant increase in the throwing rate to P3 in the EC compared with the IC (t (17) = 1.74, *p* < 0.001), indicating increased prosocial behavior while witnessing social exclusion.

Correlation analyses were also conducted between the amount of change in the throw rate between the EC and IC and empathy scores to examine whether individual differences in empathy predicted behavioral responses to social exclusion. The result indicated no significant relationship between empathy scores and behavioral change (*r* = −0.34, *p* = 0.17).

### 3.2 EEG data analysis

Data from one participant whose EEG exhibited excessive noise was excluded from EEG analysis.

To examine how EEG responses changed while witnessing exclusion, we conducted a 2×2 repeated measures ANOVA with task (WO vs. WIP) and condition (IC vs. EC) as factors separately for TBP and ABP.

**Figure 3:**
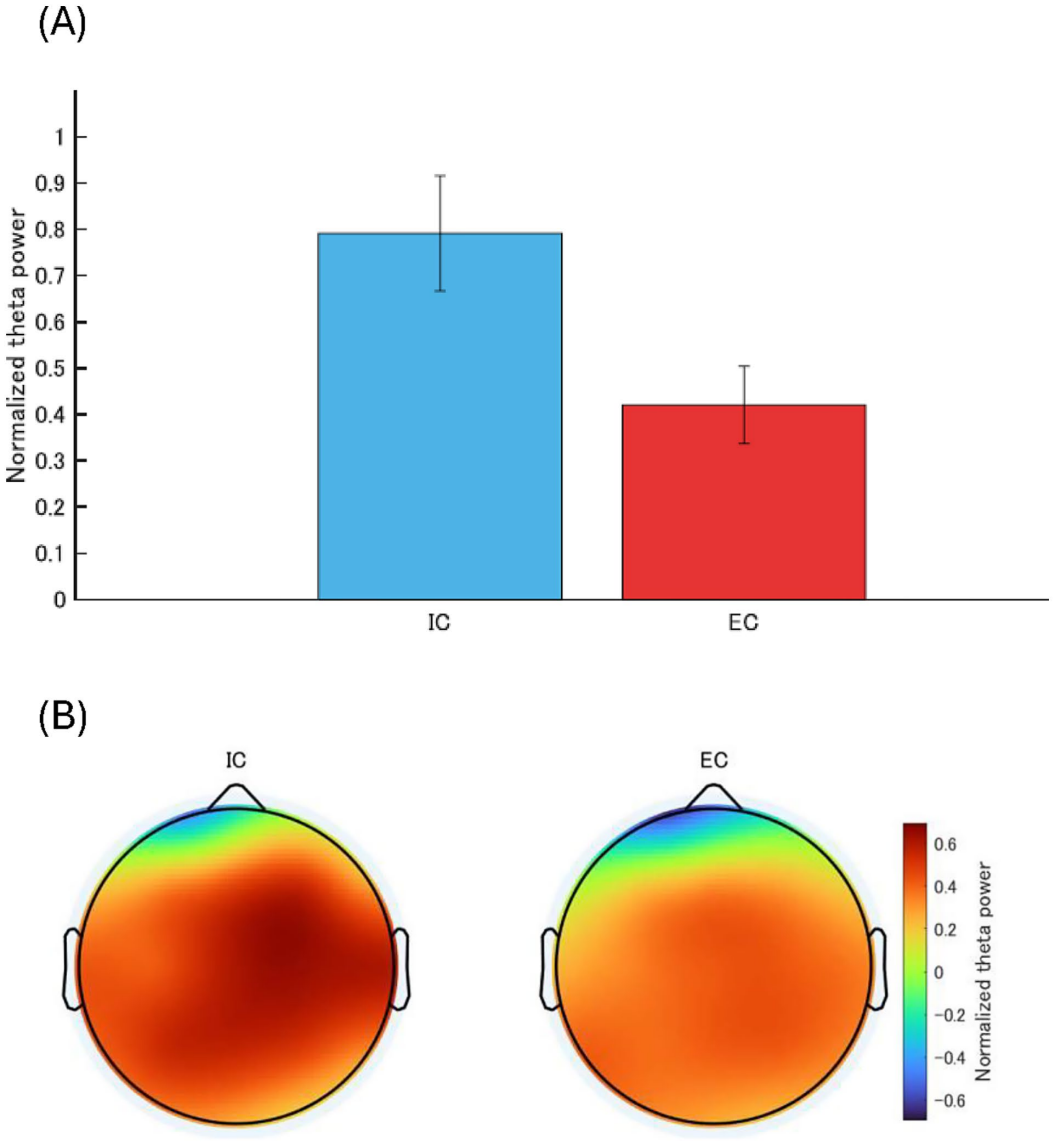
**(A)** Post-hoc result for the main effect of condition. A post-hoc test was performed using a t-test with Bonferroni correction; theta band power (TBP) is significantly lower in EC compared to IC, with error bars indicating the standard error. **(B)** TBP topographies in IC and EC conditions averaged over time periods between 500 and 1,500 ms after the onset of the throwing motion.

For TBP (Figure 3), the ANOVA results revealed a significant main effect of condition (*F* (1, 16) = 8.25, *p* = 0.011, η^2^ = 0.34) but no significant main effect of task (*F* (1, 16) = 1.37, *p* = 0.26, η^2^ = 0.08) or interaction between task and condition (*F* (1, 16) = 2.33, *p* = 0.14, η^2^ = 0.13). The post-hoc paired t-test (Bonferroni correction) showed significantly lower TBP in the EC than in the IC (t (33) = 2.68, *p* = 0.01, Cohen’s d = 0.46).

On the other hand, neither main effects nor their interaction were significant (condition: *F* (1, 16) = 0.72, *p* = 0.41, η^2^ = 0.04; task: *F* (1, 16) = 0.006, *p* = 0.94, η^2^ = 0.0004; interaction: *F* (1, 16) = 4.01, *p* = 0.06, η^2^ = 0.20). Considering the marginally significant interaction (*p* = 0.06) and the exploratory nature of investigating bystanders’ roles in prosocial intervention, we conducted a comparison examining the condition effects within each task type. Specifically, a paired ANOVA was performed for the IC/EC of WO and WIP. However, the analysis revealed no significant differences (WO: *F* (1, 16) = 3.76 *p* = 0.0.07, η^2^ = 0.19, WIP: *F* (1, 16) = 0.37, *p* = 0.55, η^2^ = 0.02).

### 3.3 Correlation between EEG responses and behavioral measures

We conducted Pearson ‘s correlation analysis between changes in throw rate and TBP and ABP during the WIP session to examine the relationship between EEG responses observed in WIP and helping behaviors. Neither TBP (*r* = −0.26, *p* = 0.29) nor ABP (*r* = −0.03, *p* = 0.90) displayed significant correlations.

## 4 Discussion

This study aimed to clarify the physiological responses of bystanders when they witness bullying. Specifically, the experiment was designed to reveal the changes in EEG responses in different situations as a bystander. Accordingly, 18 participants completed a modified Cyberball task under two sessions: Witness only (WO: not involved in bullying) and Witness in participation (WIP: involved in bullying), each containing Inclusion and Exclusion conditions.

We conducted experiments based on Hypothesis 1, which stated that observing bullying enhances empathic responses, specifically by increasing theta power and alpha suppression. The results of the ANOVA showed that, contrary to our hypothesis, there was a decrease rather than an increase in TBP during the observation of social exclusion and no significant main effect of task and task × condition interaction was observed. Thus, Hypothesis 1, which states that witnessing exclusion from P3 identifies an empathic response, was not supported. Additionally, Hypothesis 2, which stated that empathic responses would be enhanced in situations where intervention was not possible, was also not supported because the ANOVA analysis did not confirm a task (WO vs. WIP) × condition (IC vs. EC) interaction.

Although our WO session was methodologically similar to that of Masten et al. (2011), in which participants observed but did not participate in the Cyberball task, our results contradict existing findings. We hypothesized that WO would elicit stronger empathic responses than would WIP, as the inability to intervene would intensify the effect of witnessing social exclusion. However, our findings suggest that neural responses to witnessed bullying remain consistent regardless of bystander intervention opportunities. These results also raise questions about the effectiveness of the Cyberball task in eliciting empathic responses when participants serve as active bystanders rather than passive observers.

This study examined theta amplification and alpha suppression as potential neural markers of empathy, but only TBP showed significant condition-related changes. Two factors may explain the observed decrease in TBP while witnessing social exclusion.

The first factor is desensitization. Repeated exposure to cyberbullying reduces empathic response (Pabian et al., 2016) In addition, Mu et al. (2008) demonstrated that repeated exposure to others’ pain could lead to decreased upper-theta (6–8 Hz) activity. The present study suggests that continued exposure to exclusion situations causes adaptive changes in empathic responses. However, as the simple main effect of condition in ABP was not confirmed, it may be possible that empathic responses did not occur in this study’s experiment, and the decrease in TBP in EC may not reflect empathic responses. Another interpretation of this result relates to avoidance of empathy.

Witnessing bullying can be emotionally demanding, which may lead observers to inhibit their empathic responses as a protective mechanism (Zaki, 2014). A recent study by Caspar and Pech (2024) showed that obeying orders caused a decrease in frontal theta band activity, suggesting reduced cognitive conflicts in harming others through moral disengagement. These findings indicate that empathic responses may be reduced to avoid the emotional burden caused by witnessing bullying, potentially through decreased moral and cognitive conflicts. The current results indicate a reduction in frontal theta activity during EC compared to IC conditions, which may reflect this protective mechanism. However, we did not collect in-task state indices of personal distress or emotion regulation during exclusion, limiting our ability to distinguish protective down-regulation from desensitization. Future work should include brief block-level ratings and in-task emotion-regulation/physiological measures (e.g., ERQ, HRV, skin conductance) and model them as covariates/moderators. Additionally, we examined the relationship between empathic responses and behaviors. Whereas participants showed a significant increase in helping behavior toward P3, who was excluded from the game, in EC compared to IC, there was no correlation between the change in throw rate to P3 between conditions and either TBP or ABP in the WIP task. Thus, Hypothesis 3, which states that victim assistance is correlated with empathic responses, was not supported. Previous research has demonstrated that empathy is a key factor in prosocial behavior and victim support during bullying situations (Eisenberg and Miller, 1987; van Noorden et al., 2015; Riess, 2017; Barlińska et al., 2018; Valdés-Cuervo et al., 2018), suggesting a relationship between subjective empathy and bystander intervention. However, this relationship has been challenged by studies showing either no significant association between empathy and helping behaviors (Jenkins et al., 2016; Oh and Park, 2019) or even negative correlations (Barhight et al., 2013; Vachon et al., 2014). Our findings align with these latter studies, showing no significant correlation between self-reported empathy and helping behaviors. However, the small sample size (N = 18) of this study may have limited our ability to detect correlations between EEG measures (TBP and ABP) and self-reported empathy. In addition, an ERP study on vicarious ostracism focusing on compassion (Rodriguez et al., 2023) indicated an association between the posterior LPP and behavior. However, as the present study focused on the frontal region, a correlation between empathic responses and helping behaviors may not have been detected. Furthermore, although efforts were made to minimize potential influences, prior instruction may have promoted helping behaviors among participants, even if they had no intention to help. We did not add trait empathy as a covariate to the primary within-subjects ANOVAs to preserve power with *N* = 18 and because ANCOVA assumptions (e.g., homogeneity of regression slopes) are hard to verify in small samples. Future, adequately powered studies should model empathy as a covariate/moderator and include state-level measures during the task (personal distress, emotion regulation).

## 5 Limitations

This study has several important limitations that should be considered when interpreting our findings.

First, experimental design constraints might affect control over confounding factors. We could not fully control for order effects, as EC was systematically conducted after IC based on evidence that empathic responses could adapt during brief tasks (Mu et al., 2008) and that witnessing bullying may reduce empathy (Pabian et al., 2016). This fixed order may have introduced systematic biases, such as carry over effects from the preceding IC condition on the responses during EC. Additionally, the number of trials differed between WO and WIP conditions to ensure sufficient P1–P4 throws for analysis. Although rest periods were provided, fatigue can increase frontal theta activities (Wascher et al., 2014) potentially affected our findings on theta response. A two-day protocol with counter-balanced order and equalized trial counts across conditions would better control for order effects and fatigue.

Second, the small sample size (*N* = 18) represents an important limitation of this study. Post-hoc power analyses indicated that although our data had sufficient statistical power (>0.99) to detect the large differences on theta-band power, it was adequately powered only for detecting large effect sizes. The non-significant findings regarding ABP differences and correlations with behavioral changes remain inconclusive, as small-to-moderate effects could not be reliably detected. Future studies with larger sample sizes should employ mixed-effects modeling to better account for individual differences in neural and behavioral responses, as well as to evaluate the interaction between different frequency bands more comprehensively.

Third, the unbalanced gender ratio (6F/12M) limits the generalizability of our findings and precludes reliable inference about gender-related differences. Future studies should implement balanced sampling or explicitly model gender effects with sufficient statistical power to detect potential differences.

Finally, our findings pertain specifically to structurally constrained situations where intervention opportunity are absent (WO condition). These results may not directly generalize to choice-based bystander passivity that is driven by psychological factors such as low self-efficacy or lack of motivation. Future research should directly manipulate these psychological factors to understand choice-based bystander passivity.

## 6 Conclusion

Previous studies have revealed that bystanders and their empathic responses play a crucial role in bullying interventions and that bystanders adjust their behavior depending on the situation. Therefore, this study used EEG to investigate the physiological effects of the ability or inability to intervene in bullying on empathic responses.

However, contrary to previous fMRI studies, no relationship was observed between behavior and empathic responses. Concurrently, the results suggested that witnessing bullying suppresses empathic responses. These findings indicate that, regardless of whether they intervene in bullying, bystanders may reduce empathy or suppress cognitive conflict to avoid empathy, thereby alleviating self-distress.

## Data Availability

The raw data supporting the conclusions of this article will be available from the corresponding author on reasonable request.
